# Effect of Folic Acid Supplementation in Pregnancy on Preeclampsia: The Folic Acid Clinical Trial Study

**DOI:** 10.1155/2013/294312

**Published:** 2013-11-18

**Authors:** Shi Wu Wen, Josee Champagne, Ruth Rennicks White, Doug Coyle, William Fraser, Graeme Smith, Dean Fergusson, Mark C. Walker

**Affiliations:** ^1^OMNI Research Group, Department of Obstetrics and Gynecology, Faculty of Medicine, Ottawa Hospital, University of Ottawa, 501 Smyth Road, Ottawa, ON, Canada K1H 8L6; ^2^Clinical Epidemiology Program, Ottawa Hospital Research Institute, 501 Smyth Road, Ottawa, ON, Canada K1H 8L6; ^3^Department of Epidemiology, Biostatistics and Occupational Health and Department of Pediatrics, McGill University Faculty of Medicine, 3175 Cote Ste. Catherine, Montreal, QC, Canada H3T 1C5; ^4^Department of Epidemiology and Community Medicine, University of Ottawa, 451 Smyth Road, Ottawa, ON, Canada; ^5^Department of Obstetrics and Gynecology, Ste. Justine Hospital, 3175 Cote Ste. Catherine, Montreal, QC, Canada H3T 1C5; ^6^Queen's Perinatal Research Unit, Department of Obstetrics and Gynecology, Queen's University, 76 Stuart Street, Connell 4, Kingston, ON, Canada K7L 2V7; ^7^Department of Medicine, Faculty of Medicine, University of Ottawa, 501 Smyth Road, Ottawa, ON, Canada K1H 8L6

## Abstract

Preeclampsia (PE) is hypertension with proteinuria that develops during pregnancy and affects at least 5% of pregnancies. The Effect of Folic Acid Supplementation in Pregnancy on Preeclampsia: the Folic Acid Clinical Trial (FACT) aims to recruit 3,656 high risk women to evaluate a new prevention strategy for PE: supplementation of folic acid throughout pregnancy. Pregnant women with increased risk of developing PE presenting to a trial participating center between 8^0/7^ and 16^6/7^ weeks of gestation are randomized in a 1 : 1 ratio to folic acid 4.0 mg or placebo after written consent is obtained. Intent-to-treat population will be analyzed. The FACT study was funded by the Canadian Institutes of Health Research in 2009, and regulatory approval from Health Canada was obtained in 2010. A web-based randomization system and electronic data collection system provide the platform for participating centers to randomize their eligible participants and enter data in real time. To date we have twenty participating Canadian centers, of which eighteen are actively recruiting, and seven participating Australian centers, of which two are actively recruiting. Recruitment in Argentina, UK, Netherlands, Brazil, West Indies, and United States is expected to begin by the second or third quarter of 2013. This trial is registered with NCT01355159.

## 1. Introduction

Preeclampsia (PE) is a leading cause of maternal and neonatal morbidity and mortality [1, 2]. It accounts for about one-third of maternal deaths, ranking second amongst causes of pregnancy associated deaths in industrialized countries [3, 4]. A 3- to 25-fold increased risk of abruptio placentae, thrombocytopenia, disseminated intravascular coagulation, pulmonary edema, and aspiration pneumonia [5] is associated with PE. Furthermore, women with a history of PE continue to be at increased risk for future cardiovascular events [6, 7]. Since delivery is the only known cure, PE is a leading cause of indicated preterm delivery [8]. PE accounts for 25% of very low birth weight infants [9], and as many as 60% of these infants suffer from learning disabilities and are associated with a low IQ [10]. PE may also increase the risk of cardiovascular disease in the offspring through “fetal origins of adult diseases” [11, 12].

There is strong evidence from both animal and human studies, including our own large cohort studies [13, 14] to support the hypothesized protective effect of folic acid on PE. We conducted a thorough search of literature in 2008 (MEDLINE (1966—September 2013), EMBASE (1980–2013), and Cochrane Central Register of Controlled Trials (Cochrane Library 2013), using a combination of the following medical subject heading (MeSH) terms: folic acid, folate, multiple vitamin, multivitamin, gestational hypertension or hypertension in pregnancy or pregnancy induced hypertension, and PE. We identified ten relevant studies, including six cohort studies, two case-control studies, and two randomized controlled trials (RCTs). Three earlier cohort studies assessed the effect of folic acid containing multivitamins (including folic acid) and gestational hypertension (including PE) [13, 15, 16], all showed a protective effect of folic acid supplementation on PE. A recent large cohort study from Denmark also showed that regular use of folic acid in pregnancy was related to a reduced risk of PE among normal-weight women [17]. However, two recent studies in China [18] and Holland [19] failed to find an effect of folic acid supplementation on PE or gestational hypertension. A case-control study (*n* = 231 patients) in Syria did not report the crude and adjusted odds ratio (OR) [20]. Based on available data we calculated the OR as being 0.14 (95% CI = 0.06–0.31), showing a strong protective effect of folic acid supplementation. In a large case-control study in Hungary involving 1,017 pregnant women with medically recorded PE and 37,134 pregnant women without PE, Bánhidy et al. found that there was a lower risk of preterm birth of newborn infants born to pregnant women with early onset PE after folic acid supplementation from early pregnancy [21]. In a reanalysis of randomized controlled trial (*n* = 2,928 patients), the adjusted odds ratio (OR) was 0.46 (95% CI = 0.20–1.05) for the 0.2 g/day folic acid supplementation group and 0.59 (95% CI = 0.26–1.32) for the 5.0 g/day folic acid supplementation group [22].

Merchant et al. conducted a randomized trial to evaluate the effect of multivitamin (20 mg thiamine, 20 mg riboflavin, 25 mg B-6, 50 microg B-12, 500 mg C, 30 mg E, and 0.8 mg folic acid) and vitamin A supplements (30 mg beta-carotene plus 5000 IU preformed vitamin A) in relation to hypertension in pregnancy (systolic blood pressure ≥140 mm Hg or diastolic blood pressure ≥90 mm Hg at any time during pregnancy) in 955 HIV-positive pregnant Tanzanian women [23]. They found that women who received multivitamins were 38% less likely to develop hypertension during pregnancy than those who received placebo (relative risk (RR) = 0.62, 95% CI = 0.40–0.94), while no such effect was found in women who received vitamin A (RR = 1.00, 95% CI = 0.66–1.51). The result of this RCT in HIV-positive patients with folic acid as a cointervention for gestational hypertension (including PE) was quite consistent with findings from observational studies. 

In a historical cohort study, we compared the occurrence of PE between pregnant women exposed to folic acid antagonists and nonexposed women (matched by year of childbirth, type of institute at birth, and mother's residence (postal code), using the 1980 to 2000 Canadian province of Saskatchewan databases. The risks of PE (adjusted OR 1.52, 95% confidence interval (95% CI): 1.39, 1.66) and severe PE (OR: 1.77, 95% CI: 1.38, 2.28) were increased in mothers with folic acid antagonist exposure [14]. Supplementary analyses by tight matching with propensity scores restricting study participants to first and second trimester exposure and to specific categories of folic acid antagonists yielded similar results. Folic acid antagonists include a broad spectrum of drugs with a common mechanism of depleting maternal folate. Findings from the effect of maternal exposure to folic acid antagonists on the increased risk of PE add to the weight of evidence that folic acid supplementation may decrease the risk of PE.

As this review suggested, while earlier studies showed protective effect of folic acid on reduced risk of PE, some recent studies failed to find such an effect. The problem in recent studies was that nonsupplementation was rare (less than 5% in most recent studies) so that selection bias/confounding becomes difficult to control. That is why an RCT is needed to sort things out. Another issue from studies by Bodnar et al. [15] and Catov et al. [17] was that they found a beneficial effect of folic acid in lean women or normal-weight women only. We believe that this is caused by a dose issue: in the Bodnar and Catov studies most women had supplementation of 0.4 mg per day, and we proposed a dose of 4 mg per day in our FACT trial. Because of the potential genetic and metabolic defects, women with increased risk may need a higher dose. A recent study by Keating et al. found that folate uptake was decreased by amphetamine, atenolol, ethanol, ecstasy, glucose, labetalol, nicotine, and tetrahydrocannabinol [24]. Moreover, many of these drugs/substances were cytotoxic, and they differentially modulated the mRNA expression of folate placental transport systems. In our birth cohort study, we observed a dose-response relationship in high risk women (Table 1).

Before initiation of the FACT study, we cautiously assessed the potential risk of daily supplementation with 4.0 mg folic acid during pregnancy (for a duration of about 6 months) from the following four aspects: (1) short-term effects, (2) long-term effects, (3) existing policy on folic acid supplementation, and (4) health care provider support in the obstetrical community through a survey of high risk obstetricians in the country.

*(1) Short-Term Effects*. No adverse outcomes were observed in women who took very high doses of folic acid in suicide attempts [25]. No short-term adverse outcome associated with folic acid supplementation in pregnancy at the recommended dosage has been reported. In the proposed study, most of the study visits for study participants will be integrated with their routine prenatal care services. The physical and emotional burden to the participants in the proposed study is small.

*(2) Long-Term Effects*. A hypothesis found in the literature suggested that because folic acid is an essential coenzyme in purine and thymine nucleotide biosynthesis and hence DNA and RNA metabolism, it may stimulate initiation or promotion of cancers such as colorectal cancer. Findings from animal experiments and human studies of the relationship between folic acid supplementation and colorectal cancer were controversial, with some studies showing a protective effect while other studies showing a potential causative effect [26–28]. A recent meta-analysis of 10 RCTs reporting overall cancer incidence (*N* = 38, 233) gave an RR of developing cancer in patients randomised to folic acid supplements of 1.07 (95% CI = 1.00–1.14) compared to controls [29]. Meta-analyses of six RCTs reporting prostate cancer incidence showed an RR of prostate cancer of 1.24 (95% CI = 1.03–1.49) for the men receiving folic acid compared to controls, while no significant difference in cancer incidence was shown between groups receiving folic acid and placebo/control group, for any other cancer type [29]. Charles et al. followed up participants from a clinical trial of folic acid supplementation in pregnancy and found a nonsignificant increase in the risk of breast cancer deaths in the two supplementation groups (0.2 and 5.0 mg folic acid/d) as compared with placebo group [30]. This report is short and carries little description of the study population and research methodology. The number of deaths was small, the confidence intervals were wide, and the authors had no prespecified hypothesis that taking folic acid supplementation in pregnancy would increase the risk of cancer [30]. In the accompanying commentary, Oakley and Mandel suggested that the most likely explanation for the reported association was chance [31]. On the contrary, a number of other studies found that folic acid supplementation was associated with lower risk of breast cancer [31, 32]. Several more recent studies generated even more controversial results. Ebbing et al. [33] conducted a combined analysis and extended followup of participants from 2 randomized, double-blind, placebo-controlled clinical trials (a total of 6837 patients with ischemic heart disease). After a median of 39 months of treatment and an additional 38 months of posttrial observational followup, 341 participants (10.0%) who received folic acid plus vitamin B(12) versus 288 participants (8.4%) who did not receive such treatment were diagnosed with cancer (hazard ratio (HR) = 1.21; 95% confidence interval (CI) = 1.03–1.41; *P* = .02) [33]. On the other hand, in a large double-blind randomized controlled trial of 12064 survivors of myocardial infarction in secondary care hospitals in the United Kingdom between 1998 and 2008, the SEARCH Collaborative Group failed to find such an association: 678 incident cancers (11.2%) were detected in the trial arm versus 639 cases (10.6%) in the placebo arm [34]. The dose (2 mg folic acid plus 1 mg vitamin B(12) daily) was higher and the intervention was longer (6.7 years) in the SEARCH Collaborative Group study [34] versus 0.8 mg folic acid plus 0.4 mg vitamin B(12) daily and 3.2 years in the Ebbing study [33] matching placebo. Interventions in the Ebbing study were also complicated, with oral treatment with folic acid (0.8 mg/d) plus vitamin B(12) (0.4 mg/d) and vitamin B(6) (40 mg/d) (*n* = 1708); folic acid (0.8 mg/d) plus vitamin B(12) (0.4 mg/d) (*n* = 1703); vitamin B(6) alone (40 mg/d) (*n* = 1705); or placebo (*n* = 1721) [33]. Baggott et al. conducted a meta-analysis involving 6 trials (26385 patients) and found an increased cancer incidence in the folic acid-supplemented groups than the nonfolic acid-supplemented groups (relative risk = 1.21 [95% confidence interval: 1.05–1.39]) [35]. On the other hand, Clarke et al. conducted a meta-analysis of 8 large, randomized, placebo-controlled trials of folic acid supplementation involving 37485 individuals at increased risk of cardiovascular disease but did not find an increased risk of cancer: rate ratios (95% confidence intervals) were 1.05 (0.98–1.13) for overall cancer incidence, 1.00 (0.85–1.18) for cancer mortality, and 1.02 (0.97–1.08) for all-cause mortality [36]. One of the major differences between the Baggott study and the Clarke study was that Clarke et al. [36] excluded two small trials on 1955 patients with a history of colorectal adenoma while Baggott et al. included them [35]. 

The effect of long-term folic acid supplementation for cancer prevention (usually multiyears) may be quite different from the effect of short-term folic acid supplementation for PE prevention (usually a few months).

*(3) Existing Policies*. The proposed 4.0 mg folic acid supplementation in the trial arm has been recommended for women with a previous pregnancy complicated by NTDs by the federal government of Canada [37]. The recent recommendations by the Society for Obstetricians and Gynecologists of Canada (SOGC) are even more liberal in terms of dosage (5.0 mg instead of 4.0 mg) and target population (including women with other risk profiles such as epilepsy or family history or high risk ethnic group or women without obvious increased risk but with poor compliance to life-style changes for healthy pregnancy) for high dose supplementation [38].

*(4) Health Care Provider Support*. We surveyed 16 perinatologists (high risk obstetricians) in the country, and 15 of them expressed no concern of safety issue related to the trial dosage of folic acid supplementation.

In summary, our careful and thorough assessment concludes that the current data does not justify major concern of the risk of folic acid supplementation during pregnancy, and overall, the risk to benefit ratio favors conducting the trial.

## 2. Methods

This trial has been registered at http://www.controlled-trials.com/ Registration #: ISRCTN23781770 and http://www.clinicaltrials.gov/ Registration #: NCT01355159.

This trial has been approved by The Ottawa Hospital Research Ethics Board, protocol number 2009-107-01H.

## 3. The Intervention

The intervention of the FACT study is daily supplementation of 4.0 mg of folic acid from randomization until delivery of the infant. Our previous study found that about 90% pregnant women took 1.0 mg folic acid or multivitamins containing 1.0 mg folic acid on daily basis [13]. This dose of folic acid was associated with reduced risk of PE in the general population [13]. The preliminary analysis of our OaK birth cohort data demonstrated a clear dose-response relationship between folic acid supplementation and PE risk in women with additional identified risk factors (Table 1). Due to the placental, endothelial, and metabolic defects (including those of folate metabolism) leading to increased risk of developing PE, a high dose of folic acid supplementation may be required. In our birth cohort data, the limited number of women with a supplementation of >2.0 mg prevented us from further grouping them into higher dose groups; however, there is likely a continued linear relationship between folic acid dose and reduced PE risk at doses >2.0 mg. We thus propose a 4.0 mg folic acid for the trial. Women who are taking up to 1.1 mg folic acid are eligible for the trial, and since we will not ask the women to change their practice; the total dose of folic acid maybe up to 5.1 mg in the trial arm (4.0 mg from trial medication and 1.1 mg from routine supplementation) and up to1.1 mg in the placebo arm (from routine supplementation), which is consistent with SOGC's recommendation for high risk pregnancy [38]. 

### 3.1. Inclusion Criteria

Nulliparous and multiparous women:

1. ≥18 years of age at time of consent.
2. Taking ≤1.1 mg of folic acid supplementation daily at the time of randomization.
3. Live fetus.
4. Gestational age between 8^0/7^ and 16^6/7^ weeks of pregnancy (gestational age is based on the first day of the last menstrual period or ultrasound performed before 12^6/7^).
5. Planning to give birth in a participating hospital site.
6. Presenting with at least one of the following identified risk factors for PE:
  - prepregnancy chronic hypertension (or diastolic blood pressure ≥90 mm Hg on two separate occasions of at least 4 hours apart or use of antihypertensive medication for the treatment of hypertension);
  - pre-pregnancy diabetes (type I or type II);
  - twin pregnancy;
  - history of PE in the previous pregnancy;
  - BMI ≥35 kg/m^2^ within 3 months prior or during the first trimester of current pregnancy.

### 3.2. Exclusion Criteria

1. Women with known history or presence of clinically significant disease or condition which would be a contraindication to folic acid supplementation of up to 5.1 mg daily for the duration of pregnancy.
2. Women who have known major fetal anomaly or fetal demise.
3. Women who have a history of medical complications, including renal disease with altered renal function, epilepsy, cancer, or use of folic acid antagonists such as valproic acid.
4. Women who are using illicit drug or alcohol abuse (≥2 drinks per day) during current pregnancy.
5. Women with a known hypersensitivity to folic acid.
6. Women with a triplet or higher order of multiple pregnancy.
7. Women who have previously participated in this study in a previous pregnancy.

We will not exclude women who are affected by a previous NTD. These women will have a supplementation of folic acid at 5 mg daily as well. But for this indication, the supplementation will be discontinued at 12 weeks of gestation, while for the prevention of PE we propose to supplement for the whole pregnancy. 

## 4. Randomization and Blinding

A permuted blocked randomization method stratified by centre is used to allocate eligible participants. The randomization scheme is generated by an independent statistician based upon instructions from the study statistician. The randomization process consists of a computer-generated random listing of the treatment allocations stratified by centre and in variable permuted blocks of 4 and 6, due to the two groups assignment. The Method Centre at the Ottawa Hospital Research Institute has implemented randomization via the web. To ensure compliance, the trial participants are provided instruction for the appropriate use of study medication and a study treatment diary. Pill counts and review of the study treatment diary will provide data for teaching regarding compliance at each study visit to optimize results.

Participants are being randomized in a 1 : 1 ratio to 4.0 mg folic acid and placebo. For the purposes of endpoint collection, the Trial Coordinating Center, data management team, investigators, site personnel, and participants will remain blinded to whether women received the folic acid or placebo throughout the entire study. 

Folic acid 4.0 mg or placebo will be taken daily by oral administration from randomization (8^0/7^–16^6/7^ weeks) until delivery by the trial participant. We have a balanced consideration on the starting date of the intervention. Ideally, according to our hypothesis, an earlier intervention should have a better effect. However, it would not be realistic to recruit patients from participating centers earlier than 8 weeks of gestation. Both the folic acid and placebo have identical external appearances to maintain masking, and folic acid has no taste so the participants are not able to determine if they have been allocated to the treatment or placebo group. We expect that the folic acid from food intake and routine supplementation between trial arm and placebo arm will be balanced through randomization. However, we will collect information on additional sources of folic acid in concomitant medications and a food frequency questionnaire (Block Dietary Folate Equivalents (DFE) Screener, http://www.nutritionquest.com/) and will analyze these impacts on study results. 

## 5. Followups

The study participants will have 5 study visits. First visit is at recruitment between 8^0/7^–16^6/7^ weeks of gestation, second visit at 24^0/7^–26^6/7^ weeks of gestation, third visit at 34^0/7^–36^6/7^ weeks of gestation, fourth visit postpartum just after delivery, and fifth visit as a telephone interview 42 ± 3 days postpartum. If prenatal records are not included in the hospital records, the research team will contact the office of the treating physician to obtain prenatal records. Data on delivery and neonatal status will be abstracted from hospital charts after discharge. For participants who deliver in a centre other than the one initially planned, the research team will contact the medical center and obtain the delivery record to complete data collection.

## 6. Primary and Secondary Outcome Measures

PE is the primary outcome measure. PE is defined as blood pressure ≥90 mm Hg diastolic on two occasions ≥4 hours apart and proteinuria greater than 2+ on dipstick or greater than 300 mg in 24-hour urine collection or random protein-creatinine ratio ≥30 mg protein/mmol, developed in women greater than 20 weeks of gestation; or HELLP syndrome (hemolysis, serum LDH ≥600 U/L, serum AST ≥70 U/L, platelet count <100 × 109/L); or superimposed PE, defined as history of preexisting hypertension (diagnosed prepregnancy or before 20 weeks gestation) with new proteinuria. An adjudication committee comprised of experts in perinatology will blindly adjudicate for the primary outcome.

Secondary outcomes include maternal death, severe PE (PE with convulsion(s) or HELLP or delivery <34 weeks), abruptio placenta, preterm delivery, premature rupture of membranes, antenatal inpatient days, intrauterine growth restriction, perinatal mortality, spontaneous abortion, stillbirth, neonatal death, and neonatal morbidity including retinopathy of prematurity, periventricular leukomalacia, early onset sepsis, necrotising enterocolitis, intraventricular hemorrhage, ventilation, need for O_2_ at 28 days, and length of stay in neonatal intensive care unit (NICU).

## 7. Blood Pressure and Proteinuria Measurements

On visits 1, 2, and 3, systolic and diastolic blood pressure measurements will be measured in a standardized fashion by trained members of the study team, the participants' weight will be obtained on a calibrated scale, and urine for proteinuria will be evaluated by dipstick. 

## 8. Sample Size and Power Estimation

Two-sided test is assumed in the sample size calculation. Based on the literature [39], the best estimation of incidence of PE in the high risk population is 12%. With an alpha error of 5% and a power of >90%, 3,064 women (1,532 in each group) are required to demonstrate a decrease of 30% in the incidence of PE (from 12% to 8.4%) in the trial group (4.0 mg of folic acid) as compared with the placebo group [40]. We will recruit 3,656 high risk women within the study period. This will allow for noncompliance, withdrawn, loss to follow up, and other unanticipated events. The power in our study to detect 30% reduction of PE is 90%. Based on findings from observational studies, all showed unanimously a 30% or more reduction in PE in the supplementation group, suggesting that a 30% reduction in the trial arm as compared with placebo arm is achievable. Moreover, a 30% reduction would be considered clinically important. 

We anticipate a rate of loss to follow up of <10%. This estimate is a realistic estimate based on other trials that have recruited high risk pregnant women in their first trimester and followed to birth which had the same patient population, outcome ascertainment, treatment mediation, and duration and frequency of visits as the FACT study. Furthermore, these women have existing medical complications of pregnancy that require close management and allows for study visits to be combined with antepartum visits with the high risk care provider. 

## 9. Data Analyses

The analysis will be carried out on an “intention to treat” basis. We will first compare the difference in prognostic variables, compliance, and folic acid intake from other sources between intervention and placebo groups. We will then compare the outcomes between the intervention and placebo groups and make adjustment for prognostic and other factors that might confound the comparison.

Chi-square test will be used in the comparison of incidence of PE between the intervention and placebo groups. Multiple logistic regression analysis will be used to adjust for potential confounding by parity (0, ≥1, 0 as the reference), age (<20, 20–34, ≥35, 20–34 as the reference), cigarette smoking (yes, no, no as the reference), and other important prognostic factors identified at the description stage. 

Chi-square test will be used in the comparison of the occurrences of secondary outcome measures, and *t*-test will be used in the comparison of means of birth weight and gestational age, between the intervention and placebo groups. Multiple logistic regression will be used for binary outcomes, and multiple linear regression analysis will be used for continuously distributed outcomes to adjust for confounding by parity, age, cigarette smoking, and other important prognostic factors.

Interim analysis will be performed by the independent DSMB when one-half or 1,828 participants have been randomized and visit 5 (postpartum telephone interview at 42 ± 3 days) has been completed to verify the study trial assumptions. All adverse events will be collected from the time of randomization to visit 5 (postpartum telephone interview at 42 ± 3 days), the last completed study visit. An adverse event is defined as any untoward medical occurrence in a patient or clinical investigation subject administered a pharmaceutical product and which does not necessarily have a causal relationship with the treatment in accordance with GCP. 

## 10. Results

The FACT study was funded by the Canadian Institutes of Health Research in 2009, and regulatory approval from Health Canada was obtained in 2010. We have worked diligently to implement the FACT study in Canada and internationally. A summary of the progress is presented below.

1. Development and finalization of all FACT related documents including the study protocol, monitoring plan, standard operating procedures, procedure manual, and case report forms (CRFs).
2. Receipt and PK testing of study treatment.
3. Creation and implementation of an electronic web randomization and EDCS.
4. Identification and recruitment of clinical sites (21 Canadian sites, with 18 active sites up to June 15, 2013).
5. Identification and recruitment of international collaborators (7 international collaborators in Australia, Argentina, UK, Netherlands, West Indies, Brazil, and the United States).

As of June 15, 2013, 450 participants have been randomized. Additional centers from Canada, Australia, Argentina, UK, Netherlands, West Indies, Brazil, and the United States will join our recruitment efforts soon.

## 11. Discussion

**Figure 1:**
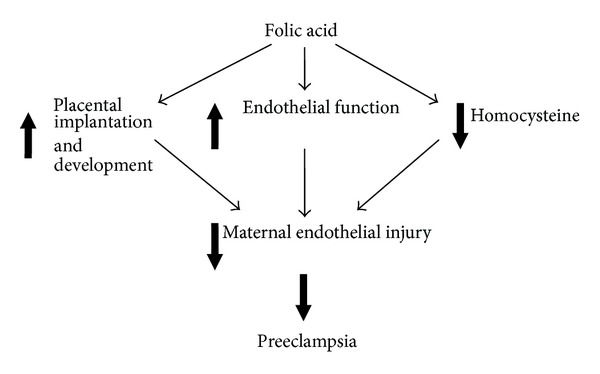
Schematic of different proposed mechanisms of action by which folic acid decreases the risk of developing preeclampsia.

The health benefit of folic acid has so far been focused on its effect on NTDs. Several observations on the association between folic acid and NTDs have been made. Examples include low folate intake levels and risk of NTDs was high in pregnancies from low socio-economic families [41, 42], mean RBC folate concentrations in women with a NTD was lower [43], folic acid metabolism in pregnant women affected by NTD was impaired [44], and the use of aminopterin, a powerful folic acid antagonist, was associated with anencephaly [45]. These observations have led to large scale randomized controlled trials of the effect of periconceptional folic acid supplementation on preventing NTDs. The trials demonstrated a dramatic effect of folic acid on NTDs, at least 70% reduction in the recurrence or first occurrence of NTDs [46, 47]. Based on evidence from the randomized controlled trials, policies and guidelines on periconceptional folic acid supplementation have been implemented since the 1990s in many countries including Canada [37, 38, 48], with high dose folic acid (4.0–5.0 mg) recommended for high risk women and low dose folic acid (0.4–1.1 mg) for low risk women in the prevention of NTDs. High dose folic acid (5 mg per day) during pregnancy to treat anaemia in earlier clinical trials [48, 49] did not show any effect on pregnancy complications, a reassurance of its safety. The hypothesis behind the effect of periconceptional folic acid supplementation on NTDs states that once the chorioallantoic placenta is formed and the fetal heart starts to perfuse it, the requirements for folic acid by the conceptus increase steeply. Based on the fact that folate is required for nucleotide synthesis and cellular methylation potential and therefore modifies DNA synthesis, cell proliferation, and gene regulation, a shortage of folate at this stage might interfere with the orderly closure of the neural tube [50]. While this mechanism may explain the observed effects of folic acid on pregnancy outcomes other than NTDs, a number of other mechanisms have been proposed to explain the observed beneficial effect of folic acid supplementation on PE (Figure 1). The first is related to placental implantation and development. A well implanted and developed placenta is essential for the health and wellbeing of the mother and the fetus. Placental growth/development is a period of increased cell proliferation and differentiation. Therefore, higher folate intakes may be required to support appropriate placental implantation and growth and development in early pregnancy. The second is related to the effect of folic acid on lowering blood homocysteine levels [51, 52], as hyperhomocysteinemia is a risk factor for a number of pregnancy complications including PE [53–55]. The third is related to the effect of folate on improving systemic endothelial function and therefore reducing the risk of such complications as PE [55–58].

The potential impacts of folic acid on maternal and child health beyond its effect on NTDs, combined with the lack of solid scientific evidence on the association between folic acid supplementation and adverse outcomes in mothers and offspring, have created a dilemma in folic acid supplementation during pregnancy. The proposed 4.0 mg folic acid supplementation in the FACT study has been recommended for women with a previous pregnancy complicated by an NTD by the federal government of Canada [37]. The recommendation by SOGC [38] is even more liberal in terms of dosage (5.0 mg instead of 4.0 mg) and of the targeted population (including women with epilepsy or family history or high risk ethnic group or women without obvious increased risk but with poor supplementation compliance) for high dose supplementation. The SOGC has recently changed their recommendation, partly because of the lack of evidence; however, many centers and physicians maintain their position on liberal use of high dose folic acid supplementation for such indications as diabetes and obesity throughout pregnancy (Dr. Mark Walker, SIOGC Chair, personal communication). If high dose folic acid is truly beneficial and more conclusive evidence of the benefit is not forthcoming, this treatment may not be offered to women at increased risk of NTDs and other adverse outcomes such as PE, thus denying future generations of women and their offspring this potentially beneficial therapy. On the other hand, if high dose folic acid supplementation is not truly beneficial and more evidence concerning lack of benefit is not forthcoming, practice may gradually change to increase the dose of folic acid supplementation, particularly because there are presently no other effective therapies to offer. Should high dose of folic acid supplementation found to be harmful, future generations of women and their offspring may suffer needlessly. Studies that can offer definitive answers to this important question are thus urgently needed.

Given the disease burden of PE, novel preventions, such as folic acid, need to undergo proper scientific investigation. The results obtained in the FACT trial will inform clinical decision making by indicating whether daily supplementation with 4.0 mg folic acid starting in early pregnancy (8 to 16 weeks of gestation) until delivery is effective in preventing PE and its associated adverse outcomes in women with increased risk of developing PE. Follow-up studies for study participants in this large trial can provide answer to the question whether folic acid supplementation during pregnancy has impact on long-term outcomes in the mothers and their offspring. 

## Figures and Tables

**Table 1:** Dose-response relationship between folic acid supplementation and PE in high risk* women, OaK birth cohort study, October 2002 to December 2005.

Dose of folic acid (mg)	No. of subjects	No. (%) PE	ORs and 95% CIs**
0	33	9 (27.27)	Reference
0.1–0.9	11	1 (9.09)	0.74 (0.06, 8.88)
1.0	186	17 (9.14)	0.32 (0.10, 1.02)
1.1–1.9	18	2 (11.11)	0.24 (0.03, 1.90)
≥2.0	34	1 (2.94)	0.08 (0.01, 0.80)

*High risk in this analysis included chronic hypertension, type 1 and type 2 diabetes, history of PE, and multiple gestation; **P < 0.05 for trend test.
